# Surgical Management of Degenerative Meniscus Lesions: The 2016 ESSKA Meniscus Consensus

**DOI:** 10.1055/s-0037-1603813

**Published:** 2017-07-28

**Authors:** P. Beaufils, R. Becker, S. Kopf, M. Englund, R. Verdonk, M. Ollivier, R. Seil

**Affiliations:** 1 Orthopaedics Department, Centre Hospitalier de Versailles, Le Chesnay, France; 2 Department of Orthopaedics and Traumatology, Hospital Brandenburg, Medical School Theodor Fontane, Havel, Germany; 3 Center for Musculosketal Surgery, Charité - University Medicine Berlin, Berlin, Germany; 4 Orthopaedics, Clinical Epidemiology Unit, Department of Clinical Sciences Lund, Faculty of Medicine, Lund University, Lund, Sweden; 5 Universiteit Gent, Ghent, Belgium; 6 Département de l'Appareil Locomoteur, Centre Hospitalier de Luxembourg - Clinique d' Eich, Luxembourg, Germany; 7 Sports Medicine Research Laboratory, Luxembourg Institute of Health, Luxembourg, Germany

**Keywords:** meniscus, degenerative lesion, arthroscopic partial meniscectomy, management, consensus

## Abstract

**Purpose** A degenerative meniscus lesion is a slowly developing process typically involving a horizontal cleavage in a middle-aged or older person. When the knee is symptomatic, arthroscopic partial meniscectomy has been practised for a long time with many case series reporting improved patient outcomes. Since 2002, several randomised clinical trials demonstrated no additional benefit of arthroscopic partial meniscectomy compared to non-operative treatment, sham surgery or sham arthroscopic partial meniscectomy. These results introduced controversy in the medical community and made clinical decision-making challenging in the daily clinical practice. To facilitate the clinical decision-making process, a consensus was developed. This initiative was endorsed by ESSKA.

**Methods** A degenerative meniscus lesion was defined as a lesion occurring without any history of significant acute trauma in a patient older than 35 years. Congenital lesions, traumatic meniscus tears and degenerative lesions occurring in young patients, especially in athletes, were excluded. The project followed the so-called formal consensus process, involving a steering group, a rating group and a peer-review group. A total of 84 surgeons and scientists from 22 European countries were included in the process. Twenty questions, their associated answers and an algorithm based on extensive literature review and clinical expertise, were proposed. Each question and answer set was graded according to the scientific level of the corresponding literature.

**Results** The main finding was that arthroscopic partial meniscectomy should not be proposed as a first line of treatment for degenerative meniscus lesions. Arthroscopic partial meniscectomy should only be considered after a proper standardised clinical and radiological evaluation and when the response to non-operative management has not been satisfactory. Magnetic resonance imaging of the knee is typically not indicated in the first-line work-up, but knee radiography should be used as an imaging tool to support a diagnosis of osteoarthritis or to detect certain rare pathologies, such as tumours or fractures of the knee.

**Discussion** The present work offers a clear framework for the management of degenerative meniscus lesions, with the aim to balance information extracted from the scientific evidence and clinical expertise. Because of biases and weaknesses of the current literature and lack of definition of important criteria such as mechanical symptoms, it cannot be considered as an exact treatment algorithm. It summarises the results of the “ESSKA Meniscus Consensus Project” ( http://www.esska.org/education/projects ) and is the first official European consensus on this topic. The consensus may be updated and refined as more high-quality evidence emerges.

**Level of Evidence** I.

## Introduction

Degenerative meniscus lesions (DMLs) develop slowly and typically involve a horizontal cleavage of the meniscus in middle-aged or older persons. They are frequent in the general population, and their prevalence increases with age, ranging from 16% in knees of 50–59 year-old women to over 50% in men aged 70–90 years. 10 Magnetic resonance imaging (MRI) will typically identify a linear intra-meniscus signal, 18 often communicating with the articular surface. This hypersignal is reported to be the result of ongoing mucoid degenerative changes. Such a DML can be considered as an ageing or degenerative process. Although there is a clear correlation between knee osteoarthritis and meniscus degeneration, it is sometimes difficult to establish a clear line of distinction between these two entities.

Arthroscopic partial meniscectomy (APM) is one of the most popular orthopaedic procedures, especially for DMLs, and its incidence has been growing in several countries. 1 26 Post-operative improvement has been reported, even for patients with a DML, 6 but some complications or failures have also been witnessed, 21 and the high risk of osteoarthritis after APM remains a concern. 27 Since 2002, the majority of randomised controlled trials (RCTs) dealing with the treatment of DMLs (except Gauffin et al 11 ) demonstrated no additional benefit of APM compared to non-operative treatment or sham surgery/sham APM at a short- and mid-term follow-up. 13 14 16 17 19 24 30 However, there is a considerable gap between clinical reality and the conclusions of these studies promoting non-operative treatment to be used as the first line of treatment in the daily clinical practice. In Denmark, for instance, the overall annual incidence of surgical meniscus procedures per 100,000 persons has doubled from 164 in 2000 to 312 in 2011. A twofold increase was found for patients aged between 35 and 55 years and a threefold increase for those older than 55 years. 26 This corresponds approximately to the same period in which the above-mentioned RCTs have been published.

Given the complex clinical reality, running RCTs can give rise to bias. 7 8 For example, patients starting out with a conservative treatment for a DML sometimes require surgery before the planned follow-up period is over. Such a change of the study makes the interpretation of the results complex and may weaken the conclusion of an RCT, despite its stronger methodological design in comparison to studies with a lower level of evidence. Nevertheless, these RCTs exist, and despite their weaknesses, they give an important message. Well-performed RCTs provide a higher level of evidence than case series or clinical impressions. The latter, for example, ignore placebo and other contextual effects always explain a variable proportion of the treatment outcomes. Bearing this in mind, the treatment of a patient with a symptomatic knee and a DML should be related both to scientific evidence and clinical expertise.

The publication of the above-mentioned RCTs introduced a big controversy in the medical community. This was emphasised by B. Reider in his editorial entitled “To cut…or not to cut:” 20 “it is not surprising that we orthopaedic surgeons like doing orthopaedic surgery…but as ethical physicians, we only want to do so when it is the best interest of our patients.” In this debate, several editorials and letters have been published. 5 9 28 These controversial exchanges have not always been useful to the clinician in his/her decision-making process concerning patients with a symptomatic knee and a DML. Therefore, there is a need for a more uniform and clear consensus. This has been underlined in a recent editorial in the KSSTA journal where we stated that “the necessity of a consensual process becomes clear, founded on the independence of the organisers and with the participation of all interested parties. Work of this kind will permit a probable reduction in the number of arthroscopic meniscal resections in our countries in favour of abstention and an improved nosological definition of “meniscectomy”, rendering it pertinent and efficient.” 4

In order to assist surgeons in their treatment indications, ESSKA has, therefore, decided to initiate a European Meniscus Consensus Project. The first part, presented here, is devoted to DMLs. The complete report of the project can be found on the Society's website ( http://www.esska.org/education/projects ). The reader is cautioned that this is not a systematic literature review on the topic of DML. In addition, this project should be considered as a “framework” rather than “strict guidelines.” Its goal was to provide a reference frame for the management of DMLs, based both on scientific literature and balanced expert opinion.

## Methods

**Fig. 1:**
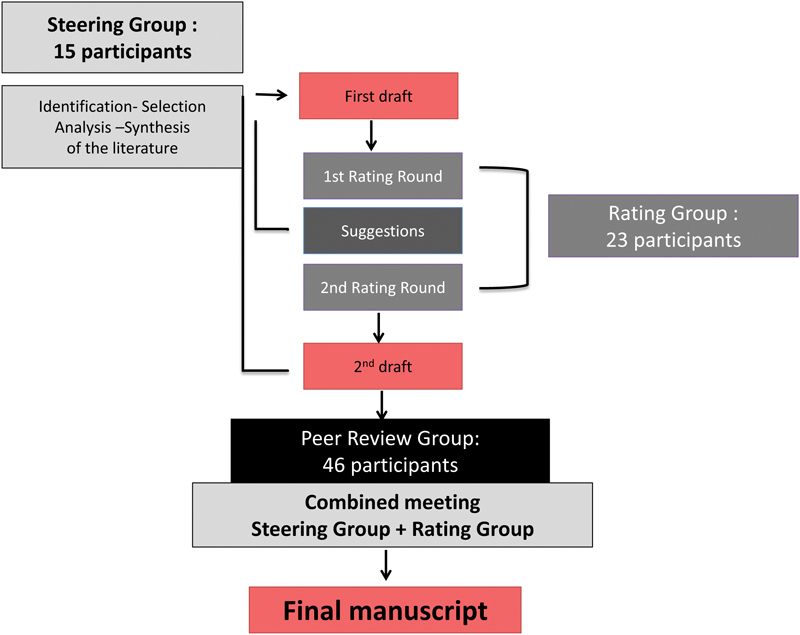
Formal Consensus Project.

In this consensus project, a DML was defined as a meniscus lesion occurring without a history of a knee trauma in a patient older than 35 years. Congenital lesions, traumatic meniscus tears and degenerative lesions occurring in young patients, especially in athletes, were excluded. The project started in December 2014, using a formal consensus process as described by the French National Healthcare Institution (Haute Autorite de Sante HAS 12 ). This process was described to be robust, clear and rigorous, as it is based on a repetitive evaluation by the following three groups of experts ( Fig. 1 ).

### The Steering Group

The steering group was composed by 15 meniscus treatment experts (13 orthopaedic surgeons, one physiotherapist and one epidemiologist) and was directed by two chairmen (PB and RB). The group had two missions: (1) Define a frame for the topic (2) Write down solid arguments based on a thorough literature review. Therefore, an extensive search of the literature was performed during January 2000–May 2015 in the following databases: PubMed, EMBASE and Medline, as well as the Cochrane Central Register of Controlled Trials (CENTRAL) (Wiley Online Library, May 2015). The searched combinations of terms included: “degenerative meniscus,” “degenerative meniscal lesion,” “degenerative meniscus surgery,” “randomised control trial,” “knee arthritis,” “lavage,” “debridement,” “clinical trial,” “meniscus imaging,” “MRI,” “horizontal cleavage,” “intrameniscus signal,” “unstable meniscus lesion,” “unstable meniscus tear,” “knee radiography,” “mechanical symptom,” “rehabilitation,” “physiotherapy,” “intra-articular injection,” “sham,” “placebo,” “hyaluronic acid,” “osteonecrosis,” “meniscectomy,” “partial meniscectomy,” “complication,” “extrusion.” Language restriction was not set in this search, and all related references were also researched. Inclusion criteria were: (1) Level I and II studies, (2) human studies, (3) published between January 2000 and May 2015 and (4) more than four patients in the treatment group. All animal or cadaveric studies and studies about revision surgeries were excluded. For topic(s) without strong scientific evidence, we included Level III and IV studies. For quality assessment, all eligible studies were evaluated independently by two reviewers (M.O. and P.B.) according to the criteria of the Cochrane Handbook for Systematic Reviews. 15 A list of questions and their related answers (question-answer sets) were defined and assorted to the levels of recommendation proposed by Shekelle et al 23 (grade A: high scientific level, grade B: scientific presumption, grade C: low scientific level, grade D: expert opinion). Both questions with limited «scientifically based answers» as well as questions with clear «scientific evidence answers» in the current literature were treated, provided that recommendations were just decreased to a lower grade.

### The Rating Group

The rating group was composed of 23 experts from 16 European countries involved in meniscus surgery in their daily practice. The mission of this group was to select and evaluate the question–answer sets through a numerical grading system. Every expert was asked to evaluate each couple by using a 1–9 points grading scale. Their recommendation was supposed to be based on the scientific level of the available literature as well as their personal experience. A value of 1 meant that the rater considered the proposal totally inappropriate (or not indicated or unacceptable), whereas a value of 9 indicated that the rater considered the proposal totally appropriate (or indicated or acceptable). Values of 2–8 represented possible intermediate situations. A proposal was deemed appropriate when the value of the median was ≥7, and the scores of each rater were ≥5. According to the formal consensus rules, low scores were not taken into account when coming from only one single rater. The proposals on which members of the rating group agreed and those on which they differed or were undecided were identified by means of votes conducted in two rounds and an interim feedback steering group meeting.

### The Peer-Review Group

This third and last group was composed of 46 orthopaedic surgeons, who perform knee arthroscopies on a daily basis and can be considered as representatives of the European community of orthopaedic surgeons who take care of painful knees. They were asked to participate in the consensus initiative through the executive boards of the affiliated national subspecialty societies of ESSKA. The mission of this group was to evaluate the manuscript draft after the grading process of the rating group in order to determine the feasibility, accessibility and readability of the proposed recommendations.

### The Manuscript Elaboration Process

After revision by the rating group, the steering group produced a manuscript which was submitted to the peer-review group. The steering group organised a final plenary assembly of both the steering and rating groups to produce a final manuscript which was submitted to the peer-review group. Finally, the steering group designed complementary documents: summary, brochure, keynote for podium presentations and scientific papers. Altogether, the complete consensus initiative involved 84 clinicians from 22 European countries. Through this long and complex process, the authors aimed at reducing the risk of any single individual or country-specific bias in the orthopaedic community and at increasing the general acceptance of the initiative due to the involvement of a large number of participants.

## Results

### The Question–Answer Sets

The question–answer sets were related to the four following subjects: the background of degenerative meniscus lesions (A), their imaging (B) and management (C), as well as a diagnostic and therapeutic algorithm (D). Background, imaging and management sections include questions, their respective answers and the proposed grade of the answers. To support each question and answer set, an extensive literature review was provided by the experts. For practical reasons, the extensive list of references (125 references) is not provided in this article. It can be downloaded from the ESSKA website ( http://www.esska.org/education/projects ).

### Results of the Grading Process

After the second rating round, the median score for each question–answer set ranged between 7.5 and 8.9. All raters scored at least five or more for each proposed question–answer couple, except one rater who scored <5 for 12 out of 20 questions. According to the formal consensus rules, these isolated low scores were not taken into account. All the question–answer sets were thus considered as appropriate.

### Background

*What is a degenerative meniscus lesion?*

**Fig. 2:**
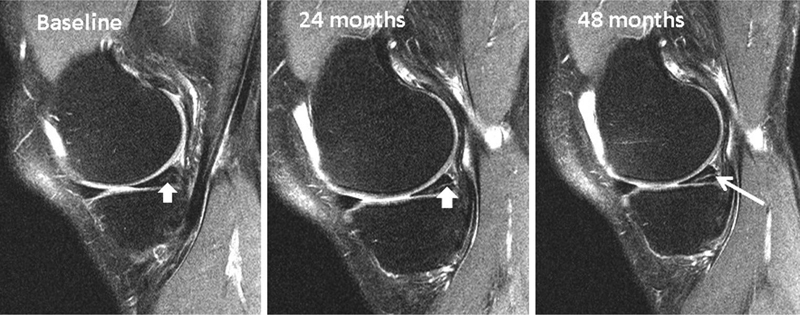
Development of an intrameniscus signal into a horizontal cleavage lesion in the posterior horn of a medial meniscus over a period of four years captured on repeat 3-Tesla knee MRI (courtesy of M Englund).

A degenerative meniscus lesion is a slowly developing lesion, typically involving a horizontal cleavage of the meniscus in a middle-aged or older person. Such meniscus lesions are frequent in the general population and are often incidental findings on knee MRI ( Fig. 2 ). The pathogenesis is not fully understood. There is often no clear history of an acute knee injury (Grade B).

*Which MRI criteria characterise a degenerative meniscus lesion?*

A degenerative meniscus lesion is usually characterised by linear intrameniscus MRI signal (including a component with horizontal pattern) often communicating with the inferior meniscus surface on at least two image slices. A more complex tear pattern in multiple configurations may also occur. The most common location of a degenerative meniscus lesion is the body and (or) posterior horn of the medial meniscus (Grade B).

*What is the prevalence of degenerative meniscus lesions?*

**Fig. 3:**
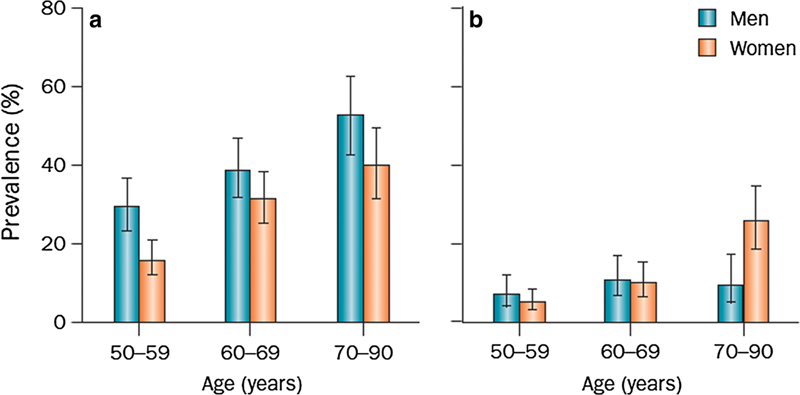
Prevalence of meniscus lesions and destruction in a randomly recruited population-based sample. ( **a** ) Meniscus tear and ( **b** ) meniscus destruction (not classified as a tear) in the right knee of men ( *n* = 426) and women ( *n* = 565) aged 50–90 from Framingham, MA, USA. The diagnosis was based on MRI. Participants were not selected on the basis of knee or other joint problems. *Error bars* show the 95% CI (reprinted with permission from New Engl J Med).

The prevalence of meniscus lesions (on the knee level) in the general population [intrameniscus signal extending to surface according to the two-slice touch rule ( Fig. 3 )] is:

1. Age 50–59 years ≈ 25%;
2. Age 60–69 years ≈ 35%;
3. Age 70–79 years ≈ 45%;
4. Patients with knee osteoarthritis ≈75–95%.

Please note that the estimates above do not include meniscus destruction/maceration, i.e. absence of normal meniscus tissue, which is also a frequent finding particularly in elderly women (Grade B).

*Do degenerative meniscus lesions cause knee symptoms?*

There is very limited evidence that pain in the degenerative knee is directly attributable to a degenerative meniscus lesion even if the lesion is considered to be unstable. Great caution must be taken before arriving at the conclusion that the degenerative meniscus lesion is the direct cause of the patients' knee symptoms (Grade B).

*Does an unstable degenerative meniscus lesion cause knee symptoms?*

While there is limited support in the literature that degenerative meniscus lesions considered to be unstable, e.g. flap tears, are truly causing knee symptoms, it is still plausible that, in some patients, torn meniscus parts from the degenerative lesion (by its displacement) may cause knee joint symptoms (Grade C).

*What are the consequences of a degenerative meniscus lesion in the knee?*

Loss of meniscus function may negatively affect the knee in the long term. Therefore, in many people, the degenerative meniscus lesion (which may impair the force transmission and load distribution capabilities of the meniscus) is a feature indicative of a knee joint with (or at increased risk of) developing osteoarthritis (Grade B).

*Are degenerative meniscus lesions a cause or consequence of knee osteoarthritis?*

**Fig. 4:**
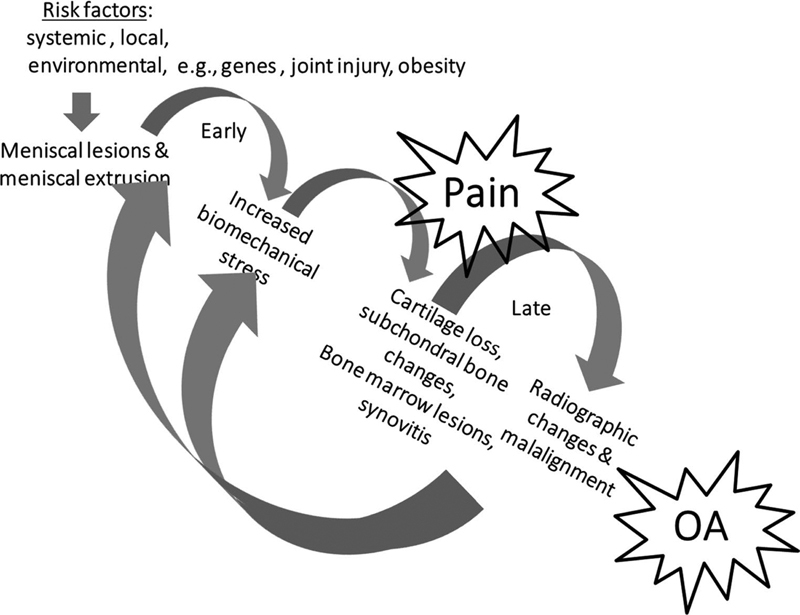
Meniscus pathway to knee osteoarthritis.

The answer to this question is still unclear. However, one causal pathway does not necessarily exclude the other, i.e. one phenotype of knee osteoarthritis may start with meniscus degradation and degenerative lesion leading to loss of meniscus function and osteoarthritis development. In turn, osteoarthritis and its general degradation of the knee joint, involving multiple structures, may also cause degenerative meniscus lesions and extrusion that further accelerate structural progression of the disease ( Fig. 4 ) (Grade B).

### Imaging

*What is the role of knee radiographs in the assessment of middle-aged or older patients with a painful knee?*

Knee radiography should be used as a first-line imaging tool to support a diagnosis of osteoarthritis or to detect certain rarer pathologies of the knee. Therefore, at least a posterior-anterior weight-bearing semi-flexed knee radiograph and a lateral view should be included in the work-up of the middle-aged or older patient with knee pain (Grade B).

*How should we make the diagnosis of knee osteoarthritis on a daily practical basis?*

The clinical diagnosis of osteoarthritis can typically be made on the basis of the duration and character of the knee joint symptoms, patient history (including the presence of strong risk factors for osteoarthritis such as age, limb malalignment, obesity, heredity, prior knee injuries and surgeries) and findings from clinical examination. In the orthopaedic setting, weight-bearing semi-flexed knee radiographs (such as the Lyon Schuss or Rosenberg view) should be included in the work-up of the middle-aged or older patient with knee pain. A skyline patella view is also important for the detection of radiographic evidence of patella-femoral osteoarthritis. Please note that plain knee radiography does not necessarily capture early stages of symptomatic knee osteoarthritis (Grade B).

*What is the role of knee MRI in the assessment of a middle-aged or older patient with a painful knee?*

Knee MRI is typically not indicated in the first-line work-up of the middle-aged or older patients with knee joint symptoms. However, knee MRI may be indicated in selected patients with refractory symptoms or in the presence of “warning flags” or localised symptoms indicating a rarer disease that needs to be ruled out, e.g. osteonecrosis. Hence, if a surgical indication is considered, based on history, symptoms, clinical exam and knee radiography, knee MRI may be useful to identify structural knee pathologies that may (or may not) be relevant for the symptoms (Grade B).

### Management

*Are functional outcomes of arthroscopic partial meniscectomy (APM) and non-operative treatment different, based on osteoarthritic (OA) status?*

No study compared OA knees with non-OA knees regarding the treatment. Thus, data are lacking on the relationship between the duration of symptoms, stage and location of OA, etc., and the treatment outcomes (Grade D) ( Table 1 ).

**Table 1:** Two RCT's specifically focused on OA knees 17 19 and five on degenerative meniscus lesions without OA: similar results

References	Inclusion criteria (arthritis)	Conclusion
Moseley et al 19	KL ≤ 4	Debridement = Sham
Kirkley et al 17	KL 2–4	Debridement = PT
Herrlin et al 13 14	Al ≤ 1	APM = PT
Katz et al 16	KL ≤ 1	APM = PT
Yim et al 30	KL ≤ 1	APM = PT
Sihvonen et al 22	KL ≤ 1	APM = Sham-
Gauffin et al 11	KL ≤ 2 + Mechanical symptoms	APM + PT > PT

Abbreviations: APM, arthroscopic partial meniscectomy; PT, physiotherapy; KL, Kellgren–Lawrence classification.

*What is the patient population defined by the RCT studies?*

Based on RCT inclusion criteria, the studies include patients with:

- Age ≥35 years (Grade A).
- Male or female (Grade A).
- Daily or almost daily knee pain >1 month (Grade A).
- Medial or lateral degenerative meniscus lesion (Grade A).
- With or without mechanical symptoms (Grade A).

*What does non-operative treatment mean?*

1. No evidence of which time/type of non-operative treatment should be proposed.
2. In the current literature, RCTs have proposed various rehabilitation protocols, however, non-operative treatment could also consist of NSAID (if no contraindications), intra-articular injection, a physiotherapy and/or home exercises for 3–6 months (Grade B).

It is important to note that no study has focused on functional outcomes of non-operative treatment versus placebo (or nothing).

*What is the rate of conversion to surgery in those patients undergoing non-operative treatment?*

Non-operative treatment is converted to surgery (crossover) in 0–35% of the patients (Grade A).

This cross-over rate has to be compared to the rate of arthroscopic treatment failure.

*Is the concept of an unstable meniscus useful for indicating meniscectomy (locking, clicking, MRI flap, etc.)?*

There are controversies regarding the definition and role of mechanical symptoms as an indication for APM. The definition of “mechanical symptoms” remains unclear and further investigations are needed, as it may cover a wide range of symptoms with different severity and frequency. In the RCT by Gauffin et al, 11 patients' history of symptoms (i.e. mechanical symptoms or acute onset of symptoms) did not affect outcomes (but patients with a joint locking lasting longer than 2s more than once a week were excluded). Pooled results of all RCTs reveal very limited added benefit of APM for degenerative meniscus regardless of pre-operative symptoms (fixed locking knee or knee with recurrent catching symptoms excluded) (Grade A).

Sihvonen et al 25 did not find any benefits over sham APM to relieve knee catching or occasional locking. (Grade A).

**Fig. 5:**
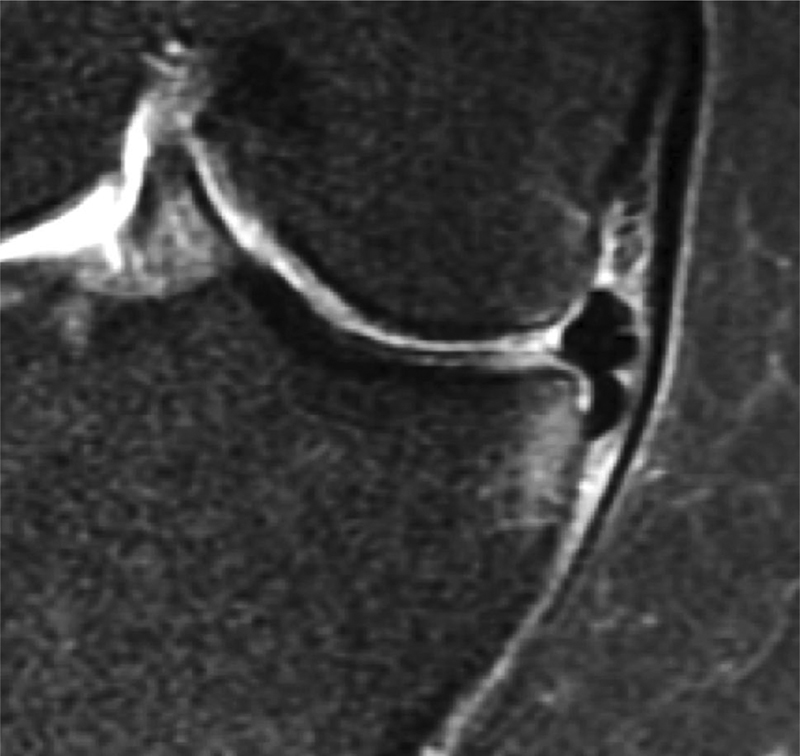
Medial meniscus flap subluxated in the tibial gutter with bony impingement. This kind of specific degenerative meniscus lesion may be associated with significant mechanical symptoms and pain.

Indication for early APM depends on the intensity and frequency of mechanical symptoms, as well as a thorough clinical examination ( Fig. 5 ) (Grade D).

*What outcomes can be expected after arthroscopic partial meniscectomy (APM)?*

**Fig. 6:**
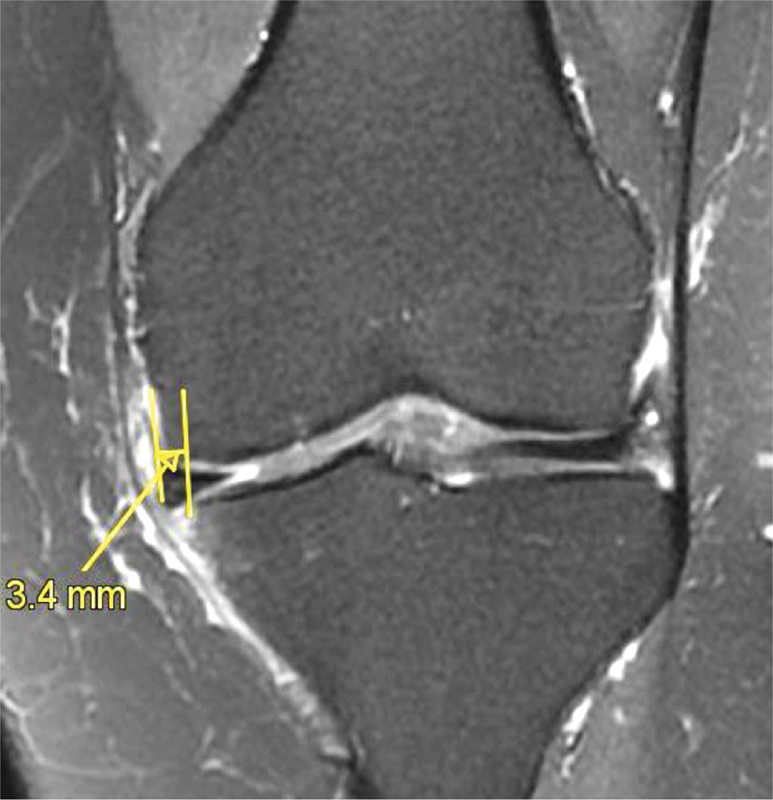
Medial meniscus extrusion (>3 mm) demonstrating an early osteoarthritic stage (MRI: coronal view; T2 FS).

1. Improvement of functional outcomes can be expected after APM (Grade A).
2. Most of the RCTs found no difference in terms of clinical outcomes after surgery compared to non-operative treatment (Grade A).
3. When surgical treatment is proposed after a non-operative treatment failure, APM will result in similar but not superior results than successful non-operative treatment (Grade A).
4. Three to six percent of patients will require another surgical procedure in the year following APM (Grade A).
5. Various predictive factors of poor results or treatment failures have been described in the current literature (increased BMI, lateral side, chondral damage, bone marrow oedema, meniscus extrusion ( Fig. 6 ) and total or subtotal meniscectomy (Grade C).

The group wants to state that:

The previous consensus statements refer to RCTs with Per-Protocol analyses. While mid-term outcomes may be similar, short-term outcomes (<12 months) might be better with APM than with non-operative treatment. The indication for early APM may also depend on the intensity and frequency of mechanical symptoms, as well as physical evaluation (Grade D).

*What is the rate of surgical complications after meniscus resection?*

The rate of surgical complication is low (0.27–2.8%) (Grade A).

After APM, the rate of complications is dependent on laterality, i.e. a lateral meniscectomy is associated with a higher rate of complications than a medial one (Grade A).

*What is the risk of osteoarthritis after meniscus resection?*

1. Patients treated with APM for degenerative meniscus lesion present a higher risk for symptomatic knee osteoarthritis compared to patients with normal knee (healthy subjects). Risk of OA is higher on the lateral side (Grade C).
2. Patients with a total meniscectomy (removal of the peripheral rim) present a higher risk for symptomatic knee osteoarthritis compared to patients with partial meniscectomy (Grade C).
3. Cartilage damage or bone marrow lesions prior to APM are major factors of poor outcomes (Grade C).
4. Meniscus extrusion ( Fig. 6 ) is associated with local osteonecrosis after APM (Grade C).

*Is there a place for arthroscopic lavage (or lavage and debridement: arthroscopic procedure including degenerative (meniscus/chondral) and/or synovial tissue debridement?) for OA knees?*

There is no place for arthroscopic lavage (or debridement) for painful knees with osteoarthritis (K/L ≥ 2). RCT's have shown that debridement/lavage has little, if any, effect on patients' short-terms reported outcomes, satisfaction or pain compared to non-operative treatment (Grade A).

Debridement might be indicated for young patients suffering from considerable mechanical symptoms (Grade D).

*When should arthroscopic partial meniscectomy (APM) be proposed?*

1. Surgery should not be proposed as a first line of treatment of DMLs (Grade A).
2. APM may be proposed after 3 months and persistent pain and/or mechanical symptoms related to a DML with normal X-rays but an abnormal MRI (Grade III meniscus lesion). The patient has to be informed about chances of successful outcomes and risks of either method (Grade B).
3. Surgery can be proposed earlier for patients presenting considerable mechanical symptoms. The patient has to be informed of chances and risks of either method (Grade D).
  However, the steering group wants to state that mechanical symptoms cannot be clearly defined according to the current literature.
4. No arthroscopic surgery should be proposed for a DML with advanced OA on weight-bearing radiographs (Grade A).

An exception should be discussed for young patients with considerable symptoms.

### Algorithm

**Fig. 7:**
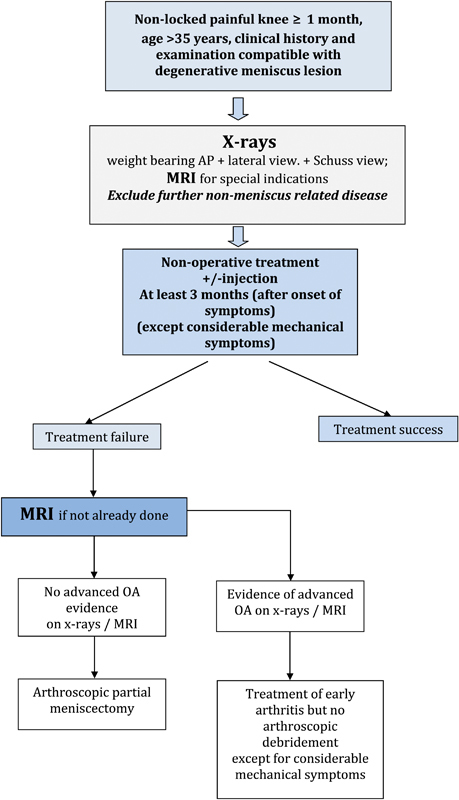
Algorithm for the management of Degenerative Meniscus Lesions.

Because of the absence of studies defining the optimal timing between the onset of symptoms, the beginning of non-operative treatment and the surgical decision following non-operative treatment failure, 3 months after the onset of the symptoms, should be considered as a reasonable delay before the decision to proceed with APM is made. This time corresponds to the mean period between non-operative treatment and conversion to APM in RCT(s) (Grade A). Three to six months should elapse after the onset of symptoms before any surgery is proposed to a patient suffering from non-locked, non-arthritic knee pain due to a DML (Grade A) ( Fig. 7 ).

Indication for surgery may be considered earlier if the patient presents with considerable mechanical symptoms (such as lack of range of motion; daily joint catching; and joint locking for more than 2s over at least 1 week) (Grade D).

## Discussion

The main finding of the European consensus in the treatment of patients with a symptomatic knee and a DML was that APM should not be proposed as a first-line treatment. The main reason is that the patient's symptoms may not necessarily relate to the actual DML but to more unspecific joint or joint line pain related to early onset osteoarthritis. APM should only be proposed after a proper standardised clinical and radiological evaluation. When investigating knee joint symptoms for a middle-aged or older patient, MRI is typically not indicated in the first-line work-up due to its high cost and the inherent and high risk of findings which are not related to the clinical problem. 10 In the daily clinical orthopaedic setting, knee radiographs should be used as an imaging tool to support a diagnosis of osteoarthritis or to detect certain rarer pathologies such as tumours or fractures of the knee.

This consensus process bears some limitations. First, we based our rationale and answers on available scientific literature: RCTs—as good as they may be—have their own biases and weaknesses. 7 RCTs including sham procedures do have a very elegant design since they eliminate the potential placebo effect of the arthroscopic procedure, but they do not correspond to daily clinical practice. Second, some clinical situations or signs are difficult to define with precision, both in the literature and in the daily clinical practice. “Mechanical symptoms” have not been exactly defined so far. They may be considered a key factor in the surgical decision-making process potentially leading to controversial conclusions. Gauffin et al 11 found better outcomes in the surgery group, independent of the presence of “mechanical symptoms” (catching, locking knee less than once a week). Sihvonen et al 25 compared outcomes of APM and sham surgery, based on the presence or absence of pre-operative mechanical symptoms. Mechanical symptoms were defined by patients' self-report as a sensation of catching or locking: true locked knees or recently locked knees were excluded. Mechanical symptoms were reported in 49% of the entire cohort. In their post hoc analysis, arthroscopic partial meniscectomy had no benefit over sham APM to relieve knee catching or occasional locking. The facts that only one-fourth of the patients showed a positive McMurray test, and conversely 49% of the patients reported mechanical symptoms, suggest that there is a need for further definition of the mechanical symptoms and description of the size, type and location of the meniscus tear. 29 In the same way, the timing to consider arthroscopic surgery can be a source of controversy. Three months from the onset of symptoms was agreed on as a general rule as it is the time generally adopted in the RCTs.

Third, a consensus, as good as it may be, is not the only factor which will influence surgeons and patients treatment decisions. There are many “peripheral” practical constraints such as the myth “I always did so, I learned to do so,” 2 the skill and simplicity of the procedure or the societal pressure (i.e. time to return to sports/work or medico-economic constraints that are highly variable between European countries and may orientate the decision in different ways). These “peripheral” constraints may limit the impact of a consensus but should not modify its main messages that non-surgical options should be the first-in-line treatment and that standardised clinical and imaging evaluation is needed before proposing an APM. Despite its inherent limitations, this work does not aim to provide a strict guideline. It should rather reflect a clear framework in the management of a DML with wellbalanced information, based on the currently available scientific evidence and the clinical expertise of 84 experienced European practitioners and scientists.

Finally, a consensus is not a final statement. It can be completed or modified with time according to the evolution of the specialty and as new evidence emerges. 3 22 As such, the present work is neither a systematic literature review, nor a formal meta-analysis, but the first European orthopaedic consensus initiative in the field of meniscus lesions. Medical professionals from a total of 22 European countries were involved in an independent and well-defined process, allowing control and feedback regarding 20 question–answer sets and an algorithm. Despite geographic and medico-economic differences among those physicians, all questions and answers eventually reached a high degree of consensus. The findings will hopefully assist every orthopaedic clinician in their decision-making when confronted with patients with a DML in a symptomatic knee.

## Conclusion

The main finding of this first European consensus in the treatment of patients with a symptomatic knee and a degenerative meniscus lesion was that arthroscopic partial menis-cectomy should not be proposed as a first-line treatment. The main reason is that the patient's symptoms are not necessarily related to the degenerative meniscus lesion, but to more unspecific pain related to early osteoarthritis. Arthro-scopic partial meniscectomy should only be proposed after a standardised clinical and radiological evaluation.

## Footnotes

a The benefit or risk of corticoid intra-articular injection has to be discussed regarding the risk of hidden osteonecrosis. Efficacy of hyaluronic acid injection is controversial.
